# Assessment of genetic purity in rice (*Oryza sativa* L.) hybrids using microsatellite markers

**DOI:** 10.1007/s13205-015-0337-y

**Published:** 2016-02-08

**Authors:** Anjana Bora, Partha Ray Choudhury, Veena Pande, Asit B. Mandal

**Affiliations:** 1 Directorate of Seed Research, Indian Council of Agricultural Research, Mau, 275101 Uttar Pradesh India; 2 Department of Biotechnology, Kumaun University, Bhimtal Campus, Nainital, 263136 Uttarakhand India

**Keywords:** Genetic purity, Grow-out-test, Hybrid rice, STMS

## Abstract

The objective of the present study is to detect genetic impurity in the seed lots of CMS lines, restorers and hybrids and to identify signature markers to differentiate parents and hybrids through DNA-based assays. Furthermore, attempts have been made to find out an alternative to Grow-Out-Test, which is very tedious, time consuming and used conventionally for seed genetic purity testing since beginning of quality seed multiplication chain. Fifty-one rice-specific sequence tagged microsatellite (STMS) primer pairs distributed throughout the rice genome were employed for fingerprinting of eight rice hybrids and their parental lines with a view to assess variation within parental lines and to test the genetic purity of the commercial seed lots. Among those, 51 markers, 28 microsatellite markers showed polymorphism (54.90 %). A total of 98 alleles were obtained with an average of 1.92 alleles per primer pair and number of alleles amplified for each primer pair ranged from 1 to 4. A set of markers were identified to differentiate parental lines of the hybrids and which emphasizes the immense scope of those molecular markers for their use in the unambiguous identification of hybrid, which would be of great benefit to farmers that depend on the hybrids.

## Introduction

Rice, *Oryza sativa* is one of the most important staple food crops in the world. About 90 % of rice is grown and consumed in Asia. The food security of more than half the world population depends upon the ability of the world to produce and supply this life-line staple food crop for our survival, sustenance and perpetuation. It has been estimated that rice production in India as well as in several other Asian countries must be doubled by 2025 A.D to meet the emerging requirements of the booming up population (Hossain 1996; Paroda 1998). Further, very recently upon considering the global climate change, it has been projected that global food production needs to be increased by 70 % by 2050 to meet the demand caused by increasing global population and thereby consumption (Varshney et al. 2011). India is considered to be leader in hybrid technology and has developed hybrids in as many as eight crops, which has boosted the productivity and production in several crops. Hybrids have a yield advantage of at least 15–20 % over high yielding inbred lines and gaining popularity in the farming community day by day being a productive input for more profitability. Hybrid technology has been exploited in many crops and poses major challenges in sustaining the benefits of hybrid technology by ensuring the genetic purity of the hybrid seeds supplied to the farmers, mostly by the private companies at very high cost. In a self-pollinated crop like rice, one of the major challenges is the production and supply of adequate quantities of pure inbred and hybrid seeds to the farmers for large-scale cultivation to augment production. It is estimated that for every 1 % impurity in the hybrid seed, the yield reduction is 100 kg per hectare (Mao et al. 1996). Maintenance of high level of genetic purity of inbred, parental lines and hybrid is thereby inevitable and indispensable to harness the benefit of high heterosis, which is the key to bring success in hybrid husbandry of any economically important crop in the public domain.

The genetic purity during multiplication stages is prone to contamination due to the presence of pollen shedders as well as physical admixtures during processing. Genetic purity test is conventionally done to assess any deviation from genuineness of the variety during its multiplications and is a compulsion for seed certification of different categories of seeds though it is very much stringent for breeder seeds since it makes the foundation of seed multiplication chain. Unambiguous identification of crop varieties and hybrids is essential for their intellectual property right (IPR) protection, prevention of unauthorized commercial use and misuse of brand name by selling spurious seeds, etc., which are rampant in India inspite of 30 regulatory rules, laws and amendments in vouge.

The purity of hybrid seed is conventionally assayed by conducting Grow-Out-Test (GOT) involving representative sample of the seed to be marketed. A set of qualitative and quantitative characters known as “descriptors” stringently related to seed quality are currently used for varietal identification. Some of the characters, particularly those showing quantitative inheritance, interact with the environment and thus make the process of variety identification empirical and sometimes illusive due to masking effect of G × E interaction. Moreover, GOT is time consuming (takes one full growing season for completion), tedious and highly vulnerable to manpower abuse and infrastructure used. Molecular markers, in contrast, being based on DNA sequence variation, provide an unbiased means of identifying crop varieties. Among the various DNA-based markers currently available, the sequence tagged microsatellite (STMS) markers are most widely used for rapid genetic purity assessment of the hybrid and parental lines (Yashitola et al. 2002; Nandakumar et al. 2004; Antonova et al. 2006; Sundaram et al. 2008 and Pallavi et al. 2011). Microsatellite markers being co-dominant in nature are preferred as desirable markers (markers of choice) in rice besides their abundance and uniform distribution throughout the genome (Akagi et al. 1996; McCouch et al. 1997). Availability of more than 2740 mapped microsatellite markers with an average density of one STMS for every 157 kb (<1 cm) of the rice genome (Cho et al. 2000; Temnykh et al. 2000; McCouch et al. 2002) has greatly improved their utility in genetic purity assessment.

The present study was undertaken to identify a set of STMS markers for fingerprinting of eight rice hybrids and their parental lines, which are being used in production en masse so that their genetic purity can be assessed and restored as well as the Sovereign right of the producers is protected by halting biopiracy. In addition, the utility of those markers was validated for the purpose of testing the genetic purity of the commercial seed lot.

## Materials and methods

### Plant materials

For the purpose of molecular identification, 8 public sector bred rice hybrids released for commercial cultivation in different parts of India in the public domain and their parental lines were obtained from rice collections maintained by rice breeders at different research institutes in India (Table 1). A random sample of 450 seeds, representing the commercial F_1_ seed lot was used for testing their genetic purity. Out of 450, 50 randomly drawn F_1_ seeds of each of the hybrids was used for analysis using rice-specific microsatellite markers and others were used for Grow-out-test (GOT). The GOT was conducted at experimental plot of Directorate of Seed Research (ICAR), Mau, Uttar Pradesh during Kharif-2009 and Kharif-2010.

**Table 1:** Details of the hybrids and their parental lines used in the study

S.No	Hybrid	Parentage		Grain character	Source
		CMS line	Restorer line		
1	PSD 1	UPRI 95-17 A	UPRI 92-133 R	Slender, long	GBPUA and T
2	PSD 3	UPRI 95-17 A	UPRI 93-287 R	Slender, long	GBPUA and T
3	CORH 3	TNAUCMS 2A	CB 87 R	Slender, medium	PBS, TNAU
4	DRRH 2	IR 68897 A	DR 714-1-2R	Slender, long	DRR, Hyderabad
5	Sahyadri	IR 58025 A	BR 827-35R	Slender, long	RARS, Karjat
6	Sahyadri 2	IR 58025 A	KJTR 2	Slender, long	RARS, Karjat
7	Sahyadri 3	IR 58025 A	KJTR 3	Slender, long	RARS, Karjat
8	Sahyadri 4	IR 58025 A	KJTR 4	Slender, long	RARS, Karjat

*GBPUA and T* Govind Ballabh Pant University of Agriculture and Technology, Uttarakhand, *PBS* Paddy Breeding Station, TNAU, Coimbatore, Tamil Nadu, *DRR* Directorate of Rice Research, Rajendranagar, Hyderabad, *RARS* Regional Agricultural Research Station, Karjat, Maharashtra

### Molecular analysis

The genomic DNA was isolated from bulked leaf samples from 5 lots each containing 10 young seedlings following Cetyl-Trimethyl Ammonium Bromide (CTAB) method (Murray and Thompson 1980). Quantification of DNA was done by analyzing the purified DNA on 0.8 % agarose gel using dilute uncut lambda DNA (400 µg/mL, Bangalore Genei Pvt. Ltd., Bangalore, India) as standard and also spectrophotometrically. The average value was used to calculate the DNA concentration. DNA was diluted in T_10_E_1_ to a concentration of about 12.5 ng/µL for PCR analysis. The sequence information for the primer pairs was obtained from the publications of Wu and Tanksley (1993), Chen et al. (1997) and Temnykh et al. (2000) and synthesized from GCC Biotech Pvt. Ltd., India.

### PCR amplification

Fifty-one STMS primer pairs (custom synthesized oligo nucleotide sequence) were selected for this study. DNA amplification was carried out in a 25 µL reaction mixture containing 1X PCR assay buffer (50 mM KCl, 10 mM Tris–Cl, 1.5 mM MgCl_2_), 200 µM each of dNTPs, 0.2 µM each of forward and reverse primers, 0.6 units of Taq DNA polymerase (Bangalore Genei Pvt. Ltd., Bangalore, India) and 25 ng of genomic DNA template. The amplification reaction was carried out in a thermal cycler (Eppendorf AG 22331, Hamburg, Germany). The first cycle consisted of denaturation of template DNA at 94 °C for 5 min, primer annealing (55 °C) for 1 min and primer extension (72 °C) for 2 min. In the next 33 cycles, the period of denaturation was reduced to 1 min while the primer annealing and primer extension time kept same as in the first cycle. The last cycle consisted of only primer extension (72 °C) for 7 min.

The amplified products were separated by electrophoresis in 3 % Metaphor™ agarose (Lonza, USA) gel containing 1 mg/ml ethidium bromide. The size of the amplified fragments was determined using size standards (Low range DNA ladder, MBI Fermentas, Lithuania). DNA fragments were visualized under UV light in a gel documentation system (Bio-Rad, USA). The markers displayed distinct amplification of specific allele combination in hybrids and parental lines and were considered as informative SSR markers. The impurities were detected based on deviation in the expected amplification pattern.

## Results

### Assessment of genetic purity through fingerprinting of rice hybrids and their parental lines

The 8 rice hybrids and their parental lines were analyzed using 51 rice-specific microsatellite markers for fingerprinting and to identify genetic impurities in the seed lot of CMS lines, restorers and hybrids. A total of 98 alleles were obtained using 51 markers with an average of 1.92 alleles per primer pair. The number of alleles amplified for each primer pair ranged from 1 to 4. The markers RM 154, RM 108 and RM 145 amplified a maximum of four alleles, while 13 STMS markers (RM 341, RM 337, RM 336, RM 144, RM 82, RM 7324, RM 177, RM 124, RM 169, RM 192, RM 3399, RM 3589, and RM 104) amplified three alleles each. Two alleles were amplified by a set of 12 markers (RM 186, RM 174, RM 269, RM 274, RM 6425, RM 297, RM 350, RM 101, RM 6881, RM 3530, RM 331, and RM 264) and rest of the 23 markers showed monomorphic (single) allelic pattern.

**Fig. 1:**
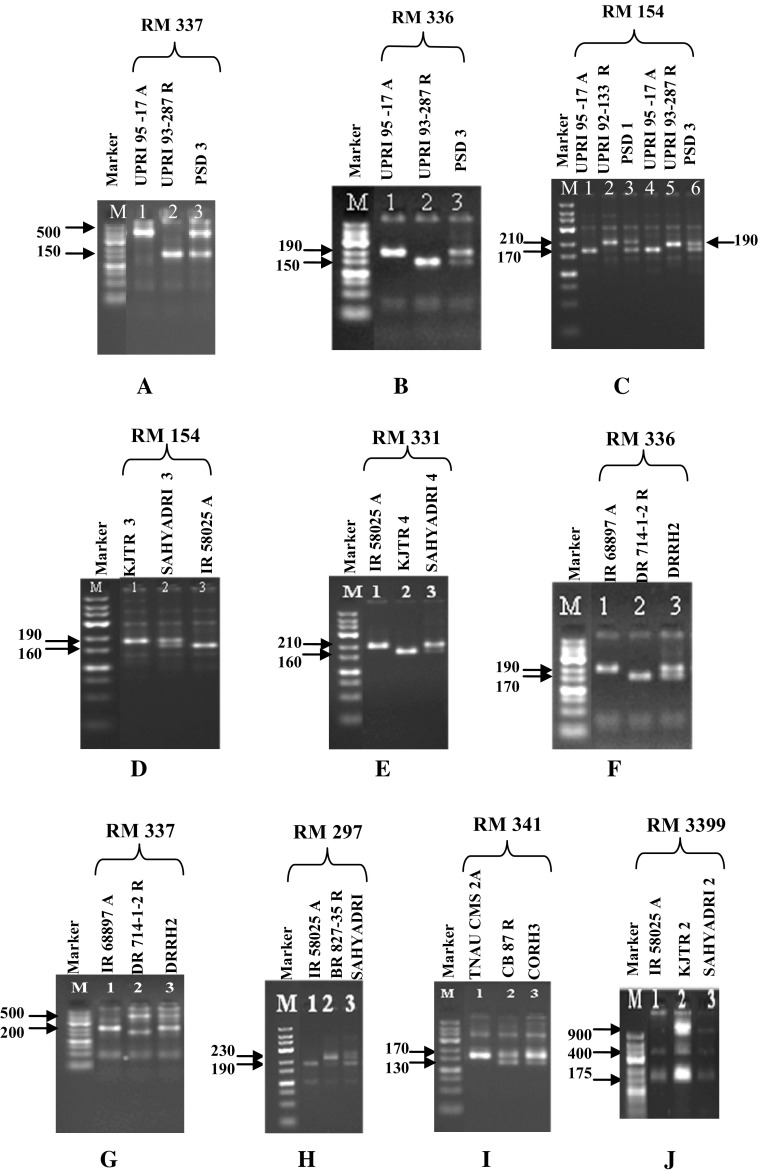
Polymorphism profile between parental lines and hybrids in rice involving microsatellite markers (the 7 selected primer pairs are represented; each lane contained bulked DNA of 10 seedlings of hybrids and their corresponding parental lines used as templates, details provided in Table 2; M: Low Range DNA Ladder, MBI Fermentas, Lithuania)

This study identified a set of seven STMS markers (RM 336, RM 337, RM 154, RM 331, RM 341, RM 297, and RM 3399), which could identify and produce unique fingerprints for all the eight rice hybrids and therefore must be considered as highly informative and may be used as molecular tags or referral markers in preparing “Molecular ID Cards” for unambiguous identification of the hybrids and their parents under reference (Table 2; Fig. 1). It was also observed that RM 101 was able to differentiate both the parents and hybrids of all the eight genotypes (hybrids and parental lines) used in the present study wherein male (300 bp), female (350 bp) and hybrid (both the amplicons) specific uniform banding pattern was obtained across the genotypes.

**Table 2:** SSR markers identified to be specific for the rice hybrids under study

Size of allele (bp)					
Hybrid	SSR marker	CL	Primer sequence (F/R)	CMS line	Restorer line
Pant Sankar Dhan 1	RM 154	2	ACCCTCTCCGCCTCGCCTCCTC CTCCTCCTCCTGCGACCGCTCC	170	210
Pant Sankar Dhan 3	RM 336	7	CTTACAGAGAAACGGCATCG GCTGGTTTGTTTCAGGTTCG	190	150
	RM 337	8	GTAGGAAAGGAAGGGCAGAG CGATAGATAGCTAGATGTGGCC	500	150
	RM 154	–		170	190
CORH 3	RM 341	2	CAAGAAACCTCAATCCGAGC CTCCTCCCGATCCCAATC	170	170, 130
DRRH2	RM 336	–		190	170
	RM 337	–		200	500
Sahyadri	RM 297	1	TCTTTGGAGGCGAGCTGAG CGAAGGGTACATCTGCTTAG	190	230
Sahyadri 2	RM 3399	4	GACGCTTCTCAACGCCAC TCTCCTCCCTCCCTCTTGTC	175, 400	175, 400, 900
Sahyadri 3	RM 154	–		160	190
Sahyadri 4	RM 331	8	GAACCAGAGGACAAAAATGC CATCATACATTTGCAGCCAG	210	160

*CL* Chromosome location, *F* Forward primer sequence (top), *R* Reverse primer sequence (bottom)

### Testing genetic purity of hybrid seeds

Characterization and identification of varieties/cultivars/genetic stocks or hybrids are crucial in any genetic improvement programme for their release in public domain and subsequent quality seed production for remunerative agriculture in the days to come. To obtain the best quality F_1_ seed in the hybrid seed production programme, high genetic and physical purity in the seeds of the parental lines are pre-requisite. Assessment of genetic purity of hybrids and their parental lines is therefore utmost important to capitalize the benefit of heterosis in any economically important crop for our better survival and prosperity.

**Fig. 2:**
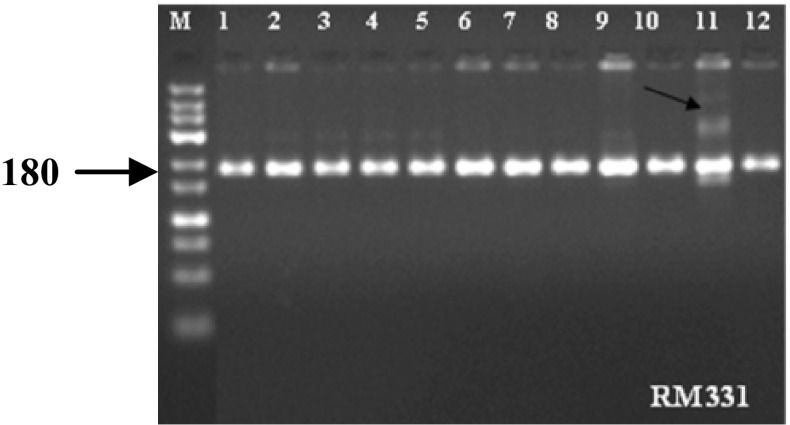
Genetic purity testing of Sahyadri 2 hybrid seeds using the STMS marker RM 331. M = Low range DNA ladder, Lane 1 = CMS line; Lane 2 = Restorer line and Lane 3–12 = individual F1 plants representing a random sample from hybrid seed lot of Sahyadri 2. Lane 11 (*arrow*) represents the off-type/contaminant

To test the genetic purity of the hybrids under study, in a random sample of 50 seeds, the marker RM 331 was found to identify an off-type in Sahyadri 2 rice hybrid (Fig. 2). This amounts to 2 % off-types in the total hybrid seed produced. The results were confirmed using 400 seeds from the same seed lot through Grow-Out-Test (GOT) in the field.

### Field performance of the rice hybrid individuals

As expected, comparative assessment of both data gleaned from the experiment involving SSR markers and GOT analyses was found to be similar *vis*-*a*-*vis* comparable. In the Grow-out trials, purity evaluation was conducted based on morphological characters including plant height and days to maturity, pollen sterility, presence of awns on the spikelets, panicle exertion, panicle length, nodal pigmentation and flag leaf senescence. The characters of few individuals that had shown deviations in Sahyadri 2 from the standard set of characters were identified as off-type and which were also further supported by the molecular marker testing.

Except Sahyadri 2, no off-type was obtained in any of the hybrids which prospects immense scope of molecular markers in genetic purity test and could be easily comparable to GOT data. In case of Sahyadri 2, however, 1/50 of the individual hybrid (2 %) was found to be off-type using molecular data. When GOT data of 400 seeds of Sahyadri 2 grown in the field were analyzed for comparison, 6 out of 400 (1.5 %) plants were detected as off-types indicating that molecular data could perform and detect off-types better than that of morphological observations. This opens a new vistas that in future the tedious painstaking and lengthy GOT test would be replaced by more precise, easy to perform genetic purity test involving molecular marker precisely with ease and confidence.

## Discussions

Determining genetic purity of the hybrids is one of the most important characteristics of good quality seed and is an essential requirement before it is sold commercially for cultivation in the field for more productivity as well as production. It is to be mentioned that there is a chance of contamination of the hybrid seed production plot through various means. Physical mixtures during the subsequent handling of the harvested materials (especially during threshing, drying, cleaning, grading, bagging) also facilitate contamination. The maintenance of high level of genetic purity of hybrid seeds is thus imperative to exploit heterosis, which is conventionally assayed by Grow-Out-Test (GOT) involving representative sample of quality seed before marketing. The GOT is essentially based on morphological (phenotypic) uniformity. The procedure is highly empirical or subjective and largely depends upon as the expression of these traits. Such qualitative and quantitative traits are greatly influenced by the environmental factors. In addition, locking up of the venture capital invested on hybrid seed production and additional expenditure incurred on storage of hybrid seed ultimately increases the hybrid seed cost. With the advent of molecular markers, these limitations of morphological markers were found to be averted confidently. The stability of microsatellite/SSR markers over different environments, do not change. Furthermore, plant stage specificity and characteristics like reproducibility, multi-allelic nature, co-dominant inheritance, relative abundance and good genome coverage (Powell et al.^1996^) make microsatellite markers a very convenient and a marker of choice for testing distinctness of varieties through assessment of genetic purity in the form of distinct amplicons profile, for future protection (Law et al. 1998) and for assessing genetic purity, specifically in high quality rice inbreds and hybrids. The use of STMS markers in hybrid rice seed purity analyses has been demonstrated earlier by many researchers. Nandakumar et al. (2004) employed 10 STMS markers for fingerprinting 11 public sector bred rice hybrids and their parental lines and tested the genetic purity of the commercial seed lot. Sundaram et al. (2008) characterized 10 each of cytoplasmic male sterile (CMS) and restorer (R) lines along with 10 popular Indian rice varieties using a set of 48 SSR markers and marker combinations, those were found unique to a particular parental line or hybrid were also identified. Likewise, rice hybrid CORH3 was identified by RM 234 (Tamilkumar et al. 2009).

In our present study, we have observed the similar banding pattern for the parental lines and hybrid of DRRH2 using RM 336 as done by Sudharani et al. (2013), which confirms the reproducibility and appropriate compatibility/suitability of microsatellites as a marker to identify hybrids and their parental lines. Similar studies have also been done by (Kumar 2012) wherein the rice hybrid DDRH 2 was identified by microsatellite markers viz. RM 204, RM 234 and RM 228.

An attempt was made in our present investigation to identify rice hybrids and their respective parents and assess genetic purity of rice hybrids using microsatellite polymorphisms. The 8 rice hybrids studied here, have been released for commercial cultivation in diverse geographical locations across India and therefore, the molecular fingerprinting of these hybrids and their parental lines warrants utmost importance for protecting the Plant Breeders’ Rights (PBR) and to halt biopiracy which is rampant in India and adjoining rice growing countries. Fifty-one STMS primer pairs selected for the study amplified a total of 98 alleles ranging from 1 to 4 alleles per primer pair with an average of 1.92 was obtained as against Tamilkumar et al. (2009) wherein 2–4 alleles per primer pair with an average of 2.9 alleles using 11 primer pairs were obtained, thus the result is comparable with the present study.

All the eight 8 rice hybrids could be precisely distinguished by the seven polymorphic STMS markers (RM 154, RM 336, RM 337, RM 341, RM 297, RM 331, and RM 3399) and unique DNA fingerprints with unique amplicons generated by those primers could be used as a set of referral STMS markers for identification of these hybrids. Therefore, it is concluded that genetic purity analysis using STMS marker may be used as a potential tool for resolving the problems arise in seed certification program owing to genetic impurity as well as the rapid determination of genetic purity of rice hybrids with the deployment of molecular makers as compared to GOT.

In a nutshell it can be concluded that use of precise DNA-based markers would perhaps replace GOT in the days to come, which is obviously very tedious, time consuming and highly vulnerable to manual mistakes that normally occur during production, processing and marketing.
